# Causes of Abnormal Ca^2+^ Transients in Guinea Pig Pathophysiological Ventricular Muscle Revealed by Ca^2+^ and Action Potential Imaging at Cellular Level

**DOI:** 10.1371/journal.pone.0007069

**Published:** 2009-09-21

**Authors:** Hiroto Nishizawa, Takeshi Suzuki, Takao Shioya, Yuji Nakazato, Hiroyuki Daida, Nagomi Kurebayashi

**Affiliations:** 1 Department of Pharmacology, Juntendo University School of Medicine, Bunkyo-ku, Tokyo, Japan; 2 Department of Cardiology, Juntendo University School of Medicine, Bunkyo-ku, Tokyo, Japan; 3 Department of Physiology, Faculty of Medicine, Saga University, Saga, Japan; University of Cincinnati, United States of America

## Abstract

**Background:**

Abnormal Ca^2+^ transients are often observed in heart muscles under a variety of pathophysiological conditions including ventricular tachycardia. To clarify whether these abnormal Ca^2+^ transients can be attributed to abnormal action potential generation or abnormal Ca^2+^ handling/excitation-contraction (EC) coupling, we developed a procedure to determine Ca^2+^ and action potential signals at the cellular level in isolated heart tissues.

**Methodology/Principal Findings:**

After loading ventricular papillary muscle with rhod-2 and di-4-ANEPPS, mono-wavelength fluorescence images from rhod-2 and ratiometric images of two wavelengths of emission from di-4-ANEPPS were sequentially obtained. To mimic the ventricular tachycardia, the ventricular muscles were field-stimulated in non-flowing Krebs solution which elicited abnormal Ca^2+^ transients. For the failed and alternating Ca^2+^ transient generation, there were two types of causes, i.e., failed or abnormal action potential generation and abnormal EC coupling. In cells showing delayed initiation of Ca^2+^ transients with field stimulation, action potential onset was delayed and the rate of rise was slower than in healthy cells. Similar delayed onset was also observed in the presence of heptanol, an inhibitor of gap junction channels but having a non-specific channel blocking effect. A Na^+^ channel blocker, on the other hand, reduced the rate of rise of the action potentials but did not result in desynchronization of the action potentials. The delayed onset of action potentials can be explained primarily by impaired gap junctions and partly by Na^+^ channel inactivation.

**Conclusions/Significance:**

Our results indicate that there are multiple patterns for the causes of abnormal Ca^2+^ signals and that our methods are useful for investigating the physiology and pathophysiology of heart muscle.

## Introduction

Action potentials generated in cardiac cells initiate Ca^2+^ influx through voltage dependent Ca^2+^ channels (VDCC). This Ca^2+^ influx, in turn, triggers the opening of ryanodine receptors in the sarcoplasmic reticulum (SR) and results in a transient elevation of cytosolic Ca^2+^ sufficient for muscular contraction. Concomitantly with this excitation-contraction (EC) coupling process, the action potentials in those cells evoke further action potentials in adjacent cells by current flux through gap junction channels. A serial propagation of this process occurs rapidly with a rate of ∼1 mm/ms at 37°C in ventricular muscle. Therefore, when Ca^2+^ signals in heart tissues are observed with a light microscope, the onset of action potential induced Ca^2+^ transients in individual cells within the field view appear synchronized.

Under a variety of pathophysiological conditions, including heart failure, ischemia-reperfusion, and ventricular tachycardia, the occurrence of Ca^2+^ transient alternans and desynchronized Ca^2+^ transients have been reported [1], [2], [3], [4], [5], [6]. The Ca^2+^ transient alternans is a phenomenon in which an alternating Ca^2+^ transient occurs from beat-to-beat. Two major mechanisms that can account for these alternans have been proposed [1], [2], [4], [7], [8], [9], [10], [11]. They can occur when action potential duration alternates from beat to beat, and the larger Ca^2+^ transient accompanies the broader action potentials. Secondly, the Ca^2+^ transient alternans can be also induced even when action potential waveforms are uniform. In this case, the amount of Ca^2+^ release from the SR alternates from beat-to-beat, i.e., the process of EC coupling is not constant [12], [13]. Furthermore, Ca^2+^ released from SR may inactivate VDCC to reduce Ca^2+^ current. Identifying how these two mechanisms interact to produce cellular alternans has remained difficult since Ca^2+^ and membrane potentials are bidirectionally coupled [2], [11].

We have been studying abnormal Ca^2+^ signals in cardiac muscles paced at ∼2 Hz in non-flowing Krebs solution [6]. The abnormal signals include delayed/desynchronized onset of Ca^2+^ transients and occasional missing Ca^2+^ transients in addition to Ca^2+^ transient alternans. The desynchronized onsets of Ca^2+^ transients, with lags of several tens of ms, were detected in some cells in a small area, 0.3 mm×0.3 mm of field of view of microscope. These observations can be explained by delayed onset of the action potentials that cause the Ca^2+^ transients, although another possible explanation would be that EC coupling was desynchronized whereas onset of action potential was synchronized.

Our preparation resembles the condition of sustained ventricular tachycardia (sustained VT) where cardiac cells are hyperactive in terms of O_2_ consumption but O_2_ supply is compromised by cardiac dysfunction [14]. Although the sustained VT can be a life-threatening arrhythmia and may easily lead to ventricular fibrillation and sudden death, the cellular activities, including membrane potential and Ca^2+^ dynamics, are not well known. It is important to determine the mechanisms of abnormal Ca^2+^ and action potential signals under such pathophysiological conditions.

Our main aim in this study was to investigate whether the abnormal Ca^2+^ responses can be attributed to abnormal action potentials or other factors such as failure of EC coupling. We monitored membrane potential and Ca^2+^ signals using the ratiometric fluorescent membrane potential indicator di-4-ANEPPS and the Ca^2+^ indicator rhod-2 in the same preparation. Our results indicate that there are multiple patterns for the causes of abnormal Ca^2+^ signals and that determination of both signals are necessary for investigation of mechanisms of cardiac arrhythmia. Causes of abnormal signals will be discussed based on effects of inhibitors of gap junctions and Na^+^ channels and of reduced current influx into isolated single cells.

## Materials and Methods

### Preparation and experimental setup

All experiments were carried out in accordance with the Guiding Principles for the Care and Use of Animals in the Field of Physiological Sciences, and approved by the Committee for Animal Experimentation of Juntendo University and the Saga University Animal Care and Use Committee. Cardiac muscle preparations were obtained following the procedure described previously [6], [15]. Briefly, guinea pigs (500∼900 g) were anaesthetized with an intraperitoneal injection of pentobarbital (100 mg kg^−1^). The heart was quickly removed from the chest and perfused via the aorta with a high-K^+^ Krebs solution. Papillary muscle bundles (0.3∼1.5 mm in diameter, 2∼6 mm in length) were excised from the left and right ventricles. For membrane potential imaging, papillary muscles were incubated in a normal Krebs solution containing 10 µM di-4-ANEPPS at room temperature for 40 min. For Ca^2+^ and membrane potential imaging in the same muscles, they were incubated with 5 µM rhod-2 AM in normal Krebs solution for 2 hr and 10 µM di-4-ANEPPS was added to the solution for the last 40 min. After washing out the dyes, the preparations were kept in Krebs solution and used within three hours.

For imaging experiments, a muscle bundle was connected with silk thread to hooks in a chamber (Warner Instruments, 8 mm in width, 30 mm in length, 2 mm in depth), stretched to its slack length, and gently pushed toward the bottom with a Plexiglas plate so that the lower surface of the bundle was 5–10 µm above the bottom. This gap allowed the cells at the lower surface access to the bathing solution. The muscle was superfused with normal Krebs solution at a rate of 2 ml min^−1^ . Experiments were carried out at 25°C.

After mounting, the muscles were conditioned by stimulating with a pair of platinum wire electrodes (0.5 mm in diameter, 15 mm in length) with current pulses of suprathreshold voltage (2 ms duration) at 0.5 Hz for >10 min. In control recording, muscles were stimulated at 0.5 or 2 Hz in flowing Krebs solution. To induce abnormal Ca^2+^ signals, ventricular muscles were stimulated at 2 Hz for 10∼20 min in non-flowing Krebs solution as described previously [6], [15].

When local stimulation was used, a pair of stimulation electrodes (0.5 mm in diameter, 3 mm in length, 2 mm separation) were placed at one end of the muscle and signals at a region 2∼3 mm from the electrodes were monitored.

### Ca^2+^ and membrane potential imaging

**Figure 1:**
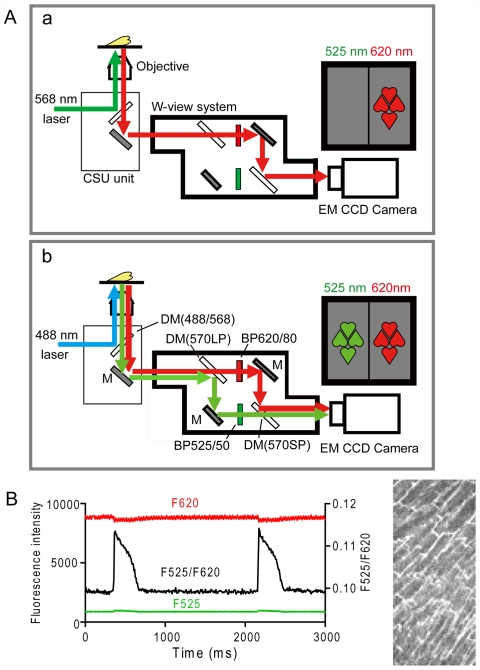
Schematic diagram of optical system (*A*) and typical ratiometric membrane potential signal (*B*). A CSU unit with a Nikpow spinning disk inside, W-view system, and EM-CCD camera are attached to the microscope (*A*). DM, dichroic mirror; M: mirror; BP, band-pass filter. *A-a* and -*b* show the same system with different selections of wavelengths. *A-a* is for determination of Ca^2+^ signal with rhod-2. *A-b* is for membrane potential signals with di-4-AENPPS. See text for details. *B*. Individual di-4-ANEPPS fluorescence signals (F525 and F620) and a ratio signal (black line) obtained from whole field of view in a cardiac muscle.

The surface cells of the papillary muscle bundles were viewed with a Nipkow disc confocal system (CSU22, Yokogawa, Japan) equipped with an Argon Krypton Ion Laser (488 and 568 nm excitation) (Figure 1A) and a 20× (Nikon, Plan Apo, N.A. = 0.75) objective lens. A W-view system (Model 8509, Hamamatsu Photonics, Hamamatsu, Japan) was used to determine the ratiometric fluorescence signal from di-4-ANEPPS [16] (Figure 1A-b). Fluorescence light from di-4-ANEPPS excited with 488 nm light was divided by a dichroic mirror (570 nm) into two components, and the fluorescence emissions at shorter and longer wavelengths were filtered through band pass-filters of 500–550 nm (BP525/50) and 580–660 nm (BP620/80) (Chroma Technology Corp., Vermont), respectively. Both fluorescent images were captured side-by-side with an EM-CCD camera (C9100, Hamamatsu Photonics, Japan) and the ratio of these images (F525/F620) were obtained (Figure 1B). For intracellular Ca^2+^ measurements, rhod-2 was excited by 568 nm and emission light was passed through the BP620/80 filter and imaged with the same camera at the one side (Figure 1A-a). The *z*-axis resolution, as estimated by imaging fluorescent beads, was 4.5 µm for the ×20 objective lens.

Data acquisition was performed using AquaCosmos software (Hamamatsu Photonics, Hamamatsu, Japan). In typical experiments, each image (0.16 mm×0.32 mm in size) was taken with 64×128 pixels every 8.7 ms (exposure time was 8 ms), or with 256×512 pixels every 17 ms (exposure time was 16 ms) and 500∼1000 images were captured during one measurement. Because di-4-ANEPPS fluorescence intensity changes produced by an action potential were small, 10% or less of total fluorescence, it was difficult to detect abnormal action potential signals by direct visual observation of fluorescence. Consequently, in this study, abnormal Ca^2+^ signals from rhod-2 were visually detected and captured as a sequence of images, and then a sequence of di-4-ANEPPS signals were obtained from the same region. Ca^2+^ signals were again monitored after the di-4-ANEPPS sequence to confirm the abnormal Ca^2+^ signals were still observed. The interval between two imaging sequences was 30 s∼1 min.

The cross-talk signal of each dye was determined. Contamination of the rhod-2 signal by the di-4 ANEPPS signals (Ex488/Em525 & Ex488/Em620) was negligible (less than 1%). Conversely, fluorescence from di-4-ANEPPS significantly increased background fluorescence of the rhod-2 signal (568Ex/620Em) depending on the loading level of di-4-ANEPPS. However, changes in membrane potential had no effect on the profile of active Ca^2+^ signals because the fluorescence change in di-4-ANEPPS due to an action potential was very small, less than 1% of the rhod-2 signal change, during a Ca^2+^ transient.

The time course of Ca^2+^ and membrane potential signals were obtained from fluorescence and ratio-signal changes from ROIs (Region of interest) which are contours of individual cells and from a whole field of view. Because ventricular cells have abundant transverse-tubule (TT) membranes, most of the membrane potential signals were assumed to derive from TT. As we did not use any reagent to inhibit muscle contractions, healthy muscles sometimes generated considerable movement during measurements. In general, Ca^2+^ signals were more affected than action potential signals by movement artifacts because of the mono-wavelength determination. Even with the movement, however, the onset of action potentials and Ca^2+^ transients could be correctly captured because they preceded muscle contractions.

### Single-cell preparation and whole-cell clamp experiments

Single ventricular cells of the guinea pig were isolated by a standard enzymatic method [17]. The cells were stored in Tyrode solution at 37°C, and were used within 8 hours after isolation. Action potentials were recorded from the cells under whole-cell clamp conditions. The cells were current-clamped through a patch pipette using a home-built patch-clamp amplifier [18], while being superfused with Tyrode solution at 37°C. Patch pipettes were fabricated from 1.5 mm thin-walled glass capillaries (No. 14-082-13, Hilgenberg) using a vertical puller (PP-83, Narishige), and a resistance of 4–6 MΩ when filled with the pipette solution. Data acquisition and analysis were done on a PC using pClamp 8 software suite (Axon) and a signal interface (DigiData 1200B, Axon) running at a sampling frequency of 20 kHz. A liquid junction potential of −8.1±1.1 mV (n = 10) was measured between the pipette solution and Tyrode solution, and was used to correct the voltage records.

### Solutions and reagents

Normal Krebs solution contained (mM): 120 NaCl, 5 KCl, 25 NaHCO_3_, 1 NaH_2_PO_4_, 2 CaCl_2_, 1 MgCl_2_ and 10 glucose and was saturated with 95% O_2_-5% CO_2_. High-K^+^ Krebs solution used for muscle preparation contained 25 mM KCl instead of 5 mM. Normal Tyrode solution used for whole cell clamp experiments contained (mM): 140 NaCl, 5.4 KCl, 1.8 CaCl_2_, 0.5 MgCl_2_, 0.33 NaH_2_PO_4_, 11 glucose, and 5 HEPES-NaOH (pH 7.4). Pipette solution contained (mM): 110 K-aspartate, 30 KCl, 10 NaCl, 5 Mg-ATP, 0.1 Tris-GTP, and 20 HEPES-KOH (pH 7.2). Di-4-ANEPPS and rhod-2 AM were obtained from Invitrogen/Molecular Probes (Eugene, OR, USA). Pilsicainide (SUN 1165), a Class Ic pure sodium channel blocker [19], [20], was provided by Daiichi-Sankyo Co. Ltd. (Tokyo, Japan).

### Data analysis

Data were analyzed using Student's t-test and reported as mean±SEM. P values below 0.05 were considered to be significant.

## Results

### Ca^2+^ and Membrane potential signals from cardiac cells in muscle paced in non-flowing solution

**Figure 2:**
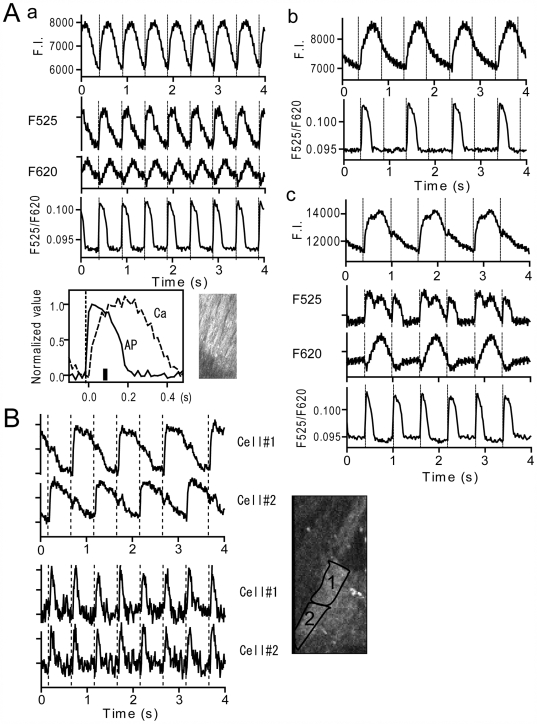
Ca^2+^ and action potential signals in muscles showing alternating Ca^2+^ transients. *A*. Signals before and during stimulation protocol in non-flowing Krebs solution in the same cardiac muscle. Upper traces in each panel are Ca^2+^ signals. Lower traces are membrane potential signals from di-4-ANEPPS. Middle traces in a and c are corresponding individual di-4-ANEPPS fluorescence signals on arbitrarily expanded scales. Stimulation was given at vertical dotted lines. *A-a*: Response to 2 Hz stimulation under control condition. *Inset* is a normalized and superimposed Ca^2+^ and action potential signal with stimulation timing matched. Motion upon stimulation started at a thick vertical bar. *A-b*: Response to 2 Hz stimulation 10 min after stopping flow of solution. *A-c*: Response to 1.67 Hz stimulation observed just after *A-b* (see Movies S1 and S2). The phases of Ca^2+^ and action potential signals in *A-b* and *A-c* were matched using the motion artifact as a clue. Note that raw fluorescence signals in this muscle contains considerable contraction-induced motion artifact. *B*. Ca^2+^ (upper panel) and action potential signals (lower panel) in two neighboring cells in a muscle after the pacing protocol. In this muscle, cell 1 and 2 showed out-of-phase Ca^2+^ transients every second stimulus whereas action potentials occurred every stimulus. This muscle showed little movement.

Figure 2A shows a series of experiments in which Ca^2+^ transients and action potential signals were sequentially determined in a muscle. Under control condition, cells in the field of view showed global Ca^2+^ transients in response to every field stimuli although a later part of the signal was contaminated with a movement artifact (Figure 2A-a upper trace). To obtain action potential signals, fluorescence images from di-4-ANEPPS at 525 and 620 nm, which increased and decreased respectively on depolarization, were captured, and the ratio was obtained as shown in Figure 1B. In the muscle shown in Figure 2A-a, however, each fluorescence signal (middle traces) was considerably distorted by movement artifact. In spite of the movement, action potential signals were reasonably well-determined (Figure 2A-a, lower trace) with a rising time (t_10–90_: a time from 10% to 90% of action potential peak) much shorter than image acquisition interval (8.7 ms). This is reasonable because the rate of rise of action potential in isolated single cells was 100∼200 mV/ms.

We have previously shown that abnormal Ca^2+^ signals in cardiac muscles could be induced by pacing at ∼2 Hz in non-flowing Krebs solution [6]. Figure S1 shows an example of the abnormal Ca^2+^ signals induced by the protocol, which was obtained in an experiments similar to Figure 3 in Kurebayashi et al. (2008) [6]. Panel A is a time-based scan image of Ca^2+^ transients in a control muscle. Cells in the muscle showed normal synchronized Ca^2+^ transients in response to the stimulation. When the muscle was stimulated at 2 Hz for 20 minutes in non-flowing Krebs solution, cells showed delayed, failed and alternating amplitudes of Ca^2+^ transients upon electrical stimuli along with a prolonged decay of Ca^2+^ transients and an elevated diastolic Ca^2+^ inferred from rhod-2 fluorescence (Panel B).

**Figure 3:**
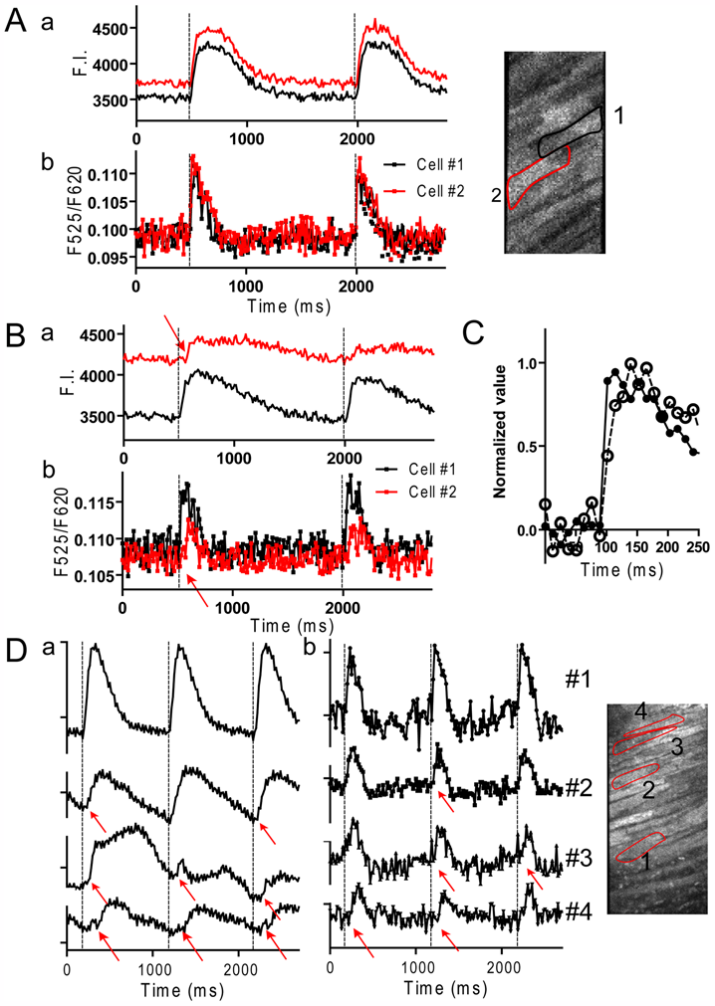
Ca^2+^ and action potential signals in muscles showing desynchronized Ca^2+^ transients. *A*. Signals under control conditions. *B*. Signals after the pacing protocol in non-flowing solution. Records in *A* and *B* were obtained from the same muscle. *C*. Overlay of the raising phase of the action potential in the whole field of view under control condition and after the pacing protocol shown in *A* and *B*. *D*. Another example of desynchronized Ca^2+^ transients and action potential signals. *a*: Ca^2+^ signals. *b*: action potential signals. Red arrows indicate delayed responses in the onset of action potentials and Ca^2+^ transients. These muscles showed little movement.

In experiments shown in Figure 2, action potentials were determined when muscle cells exhibited alternating missing of Ca^2+^ transients. In Figure 2A-b, the cardiac muscle was stimulated at 2 Hz in non-flowing Krebs solution following the control sequence shown in Figure 2A-a. After ten minutes stimulation, cells in this muscle were able to generate Ca^2+^ transients with every second stimuli. Determination of di-4-ANEPPS signal revealed that action potentials also occurred every second stimulus. This indicates that the refractory period of the myocytes was prolonged which prevented the action potentials and Ca^2+^ transients from following every stimulation.

After the 2 Hz stimulation shown in Figure 2A-b, the muscle was stimulated at lower frequency, 1.67 Hz (600 ms interval), to see whether the cells could follow a lower rate of stimulation (Figure 2A-c). At this rate, Ca^2+^ transients still occurred once every two stimuli (upper trace, see also Movie S1), but the cells were able to generate action potentials in response to every stimulus (Figure 2A-c lower trace). Movement of the muscle occurred once every two stimuli and support an alternating occurrence of Ca^2+^ transients (Figure 2A-c middle traces, Movie S2). Consequently, the failed Ca^2+^ response can be attributed to abnormal EC coupling rather than failure of action potentials. Detailed analysis of action potential signals in this muscle indicates that the duration of action potentials measured at 50% of repolarization time (APD50) with a big Ca^2+^ release (148±1.9 ms, n = 7) was ∼20% shorter than that with a lesser Ca^2+^ release (188±4 ms, n = 6). This phenomenon represents a kind of action potential-, Ca^2+^- and mechanical alternans [2], [4], [21], in that it is a severe Ca^2+^ and mechanical alternans with mild action potential alternans. In this muscle, cells in the field of view responded with synchronized Ca^2+^ transients. When the stimulation interval was further prolonged, notable alternating Ca^2+^ transients with large and small amplitudes appeared on every stimulation (Figure S2-A). This type of Ca^2+^ alternans has been described previously [1], [2], [4], [7], [13], [21], [22], [23]. On the other hand, Figure S2-B is another type of Ca^2+^ alternans that was attributed to immature action potential generation with every second stimulus.

Figure 2B is an example of two aligned cells showing alternating out of phase Ca^2+^ transients. Since this muscle moved only little upon stimulation, signals from ROIs which are contours of individual cells were determined. Determination of di-4-ANEPPS signals revealed that both cells generated action potentials. This type of Ca^2+^ alternans was less frequent than that shown in Figure 2A and primarily seen in muscles stimulated for longer times in non-flowing Krebs solution. In cases shown in Figure 2A-c and 2B, abnormal Ca^2+^ transients reflect a disorder in EC coupling rather than action potential generation.

When cardiac muscles that could generate action potentials were kept stimulated at 2 Hz or higher frequency, they often began desynchronized Ca^2+^ transients [6]. Figure 3 shows examples of delayed onset of Ca^2+^ transients. In Figure 3A, cells in this muscle initially showed synchronized Ca^2+^ transients (a) and action potentials (b) under control condition. After stimulation in non-flowing solution, cell 2 showed delayed smaller Ca^2+^ transients (Figure 3B-b). In accordance with this change, the onset of action potential signals was also delayed in cell 2 (Figure 3B-a). Figure 3D is another example of delayed initiation of Ca^2+^ transients and action potentials. The onsets in cell 4 were delayed by ∼60 ms and these abnormal Ca^2+^ transients can be attributed to distorted action potentials. Similar results were obtained in 6 preparations.

Both in Figure 3B and D, action potential signals were noisier, shorter in duration and had slower rates of rise than in normal control muscles. The rate of rise of the composite action potential in a whole field of view, which reflects the ensemble rate of rise of ∼30 cells, also became slower (Figure 3C). A rough estimate of the rate of rise of the action potential, t_10–90_, in individual cells and whole field of view are compared. The average t_10–90_ from whole field of view (37.0±2.3 ms) was significantly longer than that from individual cells (23.8±1.0 ms). This indicates that the rising phase of the action potentials was desynchronized from cell to cell in the field of view.

### Effects of gap junction inhibitor on membrane potential signals

**Figure 4:**
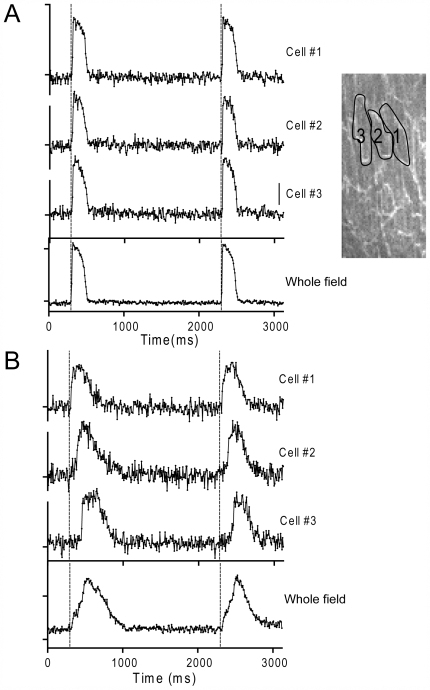
Action potential signals in the presence and absence of heptanol. *A.* Action potential signals from three adjacent cells along with the whole field of view in a control muscle. *B.* Action potential signals from same three adjacent cells and the whole field of view in the presence of 2 mM heptanol. In *A* and *B*, bars indicate ratio of 0.01.

The results in Figure 3 suggest that the generation and propagation of action potentials can be substantially suppressed in cardiac muscles paced in non-flowing solution. We have previously reported that the desynchronized onset of Ca^2+^ transients between two aligned cells could be reproduced in the presence of heptanol [6]. To examine whether such desynchronized Ca^2+^ transients could be correlated with the action potentials, action potential signals were monitored in ventricular muscles loaded with di-4-ANEPPS. Under control conditions, the onsets of action potentials were simultaneous in three aligned cells (Figure 4A upper traces) and in a whole field of view (lower trace). When heptanol at 2 mM was added to the Krebs solution, a non-simultaneous but serial onset of action potentials was detected (Figure 4B upper traces). The time lag of the onset between two aligned cells was as long as 100 ms. Similar desynchronized onsets of action potential signals were obtained with five preparations. These results indicate that delayed onset of Ca^2+^ transients seen in the presence of heptanol [6] can be attributed to delayed onset of action potentials. Supporting this, the action potential signal taken from whole field of view had a much slower rate of rise than that from individual cells (Figure 4B lower panel).

### Effect of Na^+^ channel inhibitor on the onset of action potentials

**Figure 5:**
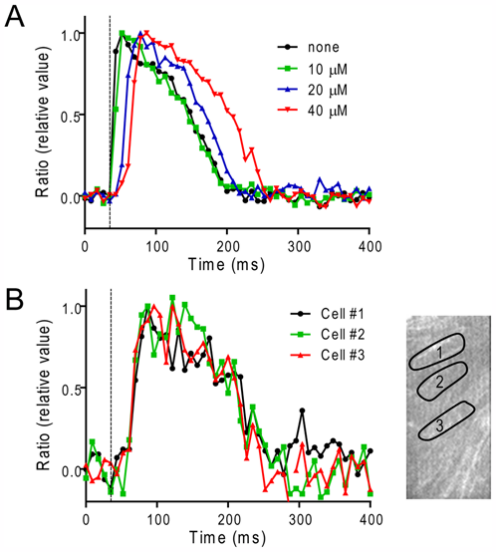
Effect of Na^+^ channel blocker on action potential signals in ventricular muscles. A healthy muscle was stimulated at 1 Hz by a pair of field electrodes placed at the end of the muscle, ∼2 mm distant from the field of view. *A.* Overlay of action potential signals from the whole field of view in the absence and presence of pilsicainide (0∼40 µM). *B.* Overlay of action potential signals in individual cells in the presence of 40 µM pilsicainide (cell 1∼3).

Another possibility for the desynchronized onset of action potentials is the inactivation of Na^+^ channels where the generation of action potentials would be substantially suppressed. Although the effects of heptanol on papillary muscles demonstrate a considerable delay in the onset of action potentials, the drug also has non-specific effects that inhibit excitability by inhibiting Na^+^ channels [24]. To assess whether the inhibition of Na^+^ channels can reproduce the delay of the action potential onset, Na^+^ channels were moderately suppressed with a Class I_C_ anti-arrhythmic, specific Na^+^ channel blocker, pilsicainide [19], [20]. For these experiments, stimulation electrodes with 2 mm separation were positioned at the end of the muscle and signals were monitored at a region ∼2 mm distant from the electrodes. Pilsicainide, at 20∼40 µM, prolonged the latency period of action potentials (Figure 5A) and Ca^2+^ transients (not shown) similarly. The prolonged latency probably reflects suppressed conduction velocity, indicating suppressed Na^+^ channel activity. In the presence of 40 µM pilsicainide, latency was ∼16 ms after stimulation, and the average t_10–90_ (27.5±6.0 ms, n = 4) was significantly slower than control (<8 ms) but similar to that in cells showing desynchronized Ca^2+^ transients (23.8±1.0 ms, n = 6). In spite of the delayed onset of action potentials, action potentials in all cells were synchronized within a field of view of 0.3 mm×0.15 mm (Figure 5B) as revealed by lack of difference between the rates of rise in individual cells (t_10–90_ = 27.5±6.0 ms, n = 4) and in the whole field of view (27.3±8.1 ms, n = 4).

**Figure 6:**
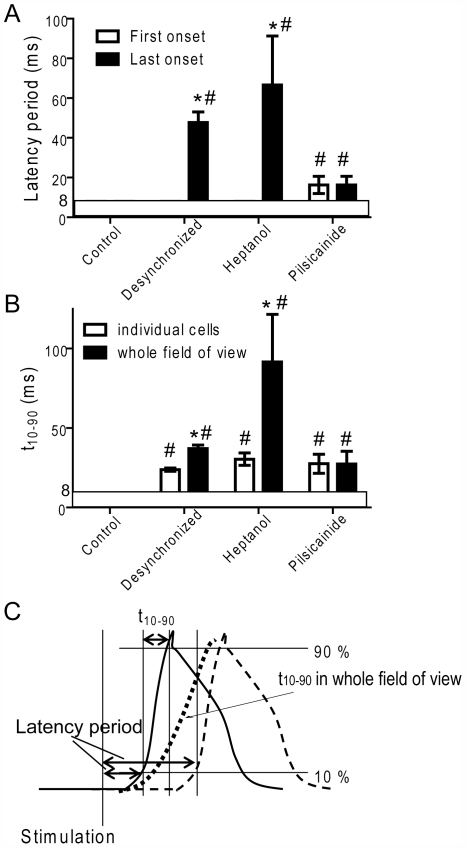
Latency (*A*) and the rate of rise (*B*) of the action potential under various conditions. *Control*: control muscles (n = 22); *Desynch*: muscles showing visually discernable desynchronization in Ca^2+^ transients (>40 ms) after the pacing protocol (n = 6); *Heptanol*: with 2 mM heptanol (n = 5); *Pilsicainide*: with 40 µM pilsicainide, muscles were stimulated locally (n = 4). *A.* Action potential latencies in cells with the first (open column) and last onsets (filled column) in the field of view were determined. *significant difference from the first onset. #significant difference from control. *B.* Rates of rise of the action potentials (t_10–90_) in randomly chosen individual cells (mean of 10 cells for each preparation were averaged for *n* preparations, open column) and in the whole field of view (filled column). *significant difference from individual cells. #significant difference from control. Data are mean±S.E.M. P<0.05. Note that the responses faster than ∼8 ms were not distinguishable. *C*. A schematic illustration of determinations of the action potential latency and the rate of rise of the action potential.

Figure 6 summarizes the latency (A) and the rate of rise of the action potentials in individual cells and whole field of views (B) under various conditions. The average t_10–90_ in individual cells was significantly reduced in muscles after the pacing protocol, in the presence of heptanol and pilsicainide. However, significant differences in the t_10–90_ between individual cells and whole field of view were detected in muscles with desynchronized onset of Ca^2+^ transients and with heptanol, but not in the presence of pilsicainide. These results indicate that the inhibition of Na^+^ channels alone could not cause desynchronized onset of action potentials.

### Reproduction of delayed onset of action potentials by small amount of current injection in isolated single cells

**Figure 7:**
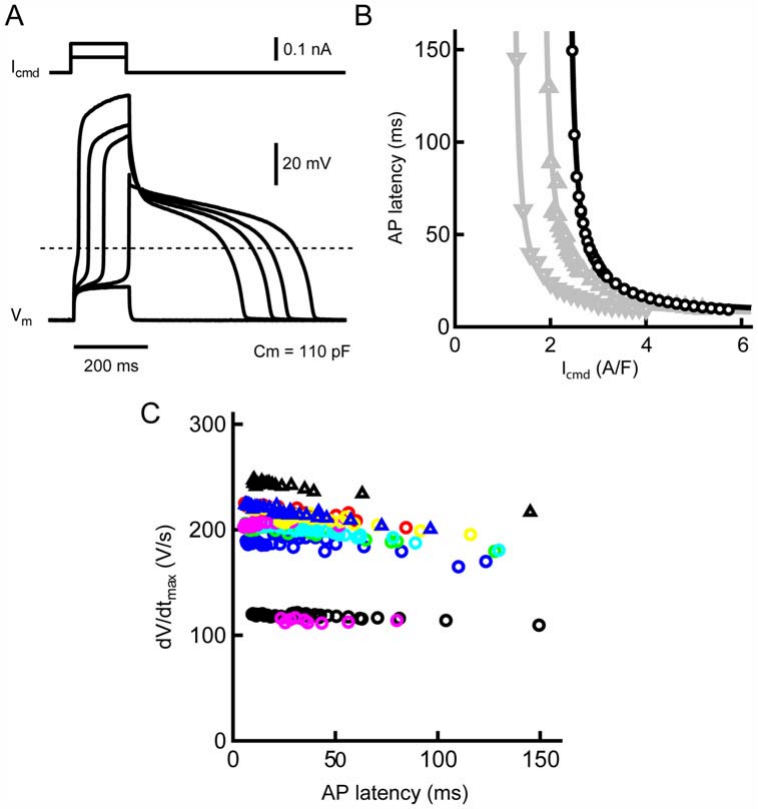
Effects of reduced depolarizing currents on action potential onset in isolated single myocytes. *A.* Typical traces of action potentials (lower records) evoked by a small command current (upper traces). *B.* Relationship between action potential latency and command current amplitude. Different symbols correspond to different myocytes. C. Relationship between the rate of rise of action potential (dV/dt_max_) and action potential latency. Different symbol correspond to different myocytes.

The results shown above indicate that the desynchronized onset of action potentials can be attributed to the suppression of gap junction channels which causes a decrease in depolarizing current influx from neighboring cells. To evaluate the possibility that reduced depolarizing current influx can mimic a slow onset of action potential generation, isolated single cardiac myocytes were injected with a very small amount of current. As shown in Figure 7A, a reduction in depolarizing current amplitude resulted in a longer latency to the onset of the action potential. Figure 7B illustrates the relationship between depolarizing current and action potential latency. Smaller depolarizing currents produced action potentials with longer latency, especially near the threshold-level. It is also noteworthy that the absolute amplitudes of the command currents required for action potential generation varied from cell to cell. Figure 7C shows the dependence of maximal rate of rise of the action potential (dV/dt_max_) on action potential latency. The dV/dt_max_ values were independent of latency period and remained relatively constant in individual cells although their absolute values were variable among cells. It is demonstrated that a delay of 100 ms or more in action potential generation can occur in association with decreased current influx into myocytes with little change in dV/dt_max_.

## Discussion

To clarify the cause of abnormal Ca^2+^ transients in pathophysiological cardiac muscle, we carried out serial measurements of Ca^2+^ and membrane potential signals in the same cardiac muscle preparations. In general, there were two reasons for abnormal Ca^2+^ transients, i.e., abnormal action potential generation and abnormal EC coupling. Determination of both signals at the cellular level is important for the investigation of the physiology and pathology of heart muscles.

### Advantage and disadvantage of serial determination of rhod-2 and ratiometric di-4-ANEPPS signals

Attempts to capture Ca^2+^ and membrane potential signals simultaneously from large numbers of pixels in the same heart tissue have been made by several groups [1], [9], [25]. The combinations of Ca^2+^ indicator and potentiometric dye that have been used are, indo-1 and di-4-ANEPPS [25] and rhod-2 and RH-237 [1], [9], but with these systems, it is difficult to take signals from individual cells. Recently Fujiwara et al. performed simultaneous recording of Ca^2+^ and membrane potential signals at the cellular level in rat hearts using RH237 and fluo-4 [26]. They utilized a dual-view, rapid-scanning confocal system, which is similar to the W-view system, and captured mono-wavelength RH237 (568 nm excitation/>665 nm Emission) and fluo-4 (488 nm excitation/535 nm emission) fluorescence signals with the same CCD camera. Although their method has the important advantage that both signals can be obtained simultaneously, a reagent like Cytochalasin D have to be used to prevent movement artifact. We used di-4-ANEPPS and rhod-2 to investigate the relation between membrane potential and Ca^2+^ signals. Compared to mono-wavelength determination, ratiometric signals of two wavelengths of emission from di-4-ANEPPS has higher signal-to-noise ratio without using a smoothing filter as noise from the light source tends to be cancelled [16], [27]. This method enabled us to distinguish cell-to-cell differences in the latency and the rate of rise of action potentials in multicellular preparations. Moreover, the ratiometric determination is more tolerant of movement artifact and reasonable signals can be obtained from moving preparations. Although Ca^2+^ and action potential signals could not be measured simultaneously with this procedure, we were able to get insight into relation between Ca^2+^ and membrane potentials because, in most cases, muscle conditions were relatively stable for several minutes, which was longer than the period of determination of both signals.

### Causes of failed Ca^2+^ transients

#### Failed action potential generation

The alternating suppression of the action potential generation indicates that cardiac cells are less responsive to triggering stimulation. The decreased responsiveness can be explained by partial inactivation of Na^+^ channels which reduces the rate of rise of the action potentials. One probable mechanism is suggested by the response of the cells to elevated [Ca^2+^]_i_ [28], [29]. In muscles used in this study, [Ca^2+^]_i_ was considerably higher than in normal muscles and the decay time of Ca^2+^ transients was significantly prolonged[6], [15]. Therefore, the Ca^2+^ extrusion by Na^+^-Ca^2+^ exchanger would be enhanced and would lead to depolarization of the resting membrane potential, which, in turn, would result in inactivation of Na^+^ channels. In addition to this, activation of K_ATP_ channels also may be involved in the reduced excitability because APD tended to be shortened after the pacing protocol [30], [31].

#### Abnormal EC coupling

The instances shown in Figure 2A-c and B are a stage where cells were able to generate action potentials at every stimulation whereas Ca^2+^ transients were observed once every two stimuli. This is an extreme case of Ca^2+^ alternans, where a large and small amplitude of Ca^2+^ transients take turn from beat-to-beat. The mechanisms of the Ca^2+^ alternans is explained by depletion of diastolic SR Ca^2+^ content after the big Ca^2+^ release and/or fluctuation of Ca^2+^ releasing activity from RyR2 which is regulated by luminal SR Ca^2+^ [1], [2], [4], [7], [12], [13]. It is interesting that muscle cells would gain Ca^2+^ during action potential generation even when Ca^2+^ transients and contractions were missed every two stimuli.

There were two patterns for the alternating missing of Ca^2+^ transients; (1) individual cells take turns with independent, out-of-phase type Ca^2+^ transients and (2) cells in a cluster show synchronous alternating Ca^2+^ transients. The latter case would contribute more to mechanical alternans. The reason for the synchronized alternating Ca^2+^ transients is unclear, but a probable explanation is that once a failure of action potential generation occurs, it may reset the Ca^2+^ release mode in all the cells to the big release with high luminal Ca^2+^. Further study is needed to examine this hypothesis.

Although we sometimes observed two-aligned cells taking turn showing alternating out-of-phase Ca^2+^ transients [6]), we never observed out-of-phase action potentials in them. Instead, cells in a cluster showed more or less concordant action potential signals. This is reasonable because action potentials tend to be coordinated by their regenerative property and by electrical coupling via gap junction channels.

### Delayed generation of action potentials

Where delayed onset of Ca^2+^ transients were detected, a similar delay was also detected in action potential signals in the same cells, indicating that the delayed Ca^2+^ transient onset is attributed to slow onset of action potential. Because the duration of the field stimulation pulse was 2 ms, action potentials occurring with several tens of ms delay could not have been induced directly by the stimulation. Instead, they must have resulted from propagated action potentials from adjacent cells. This phenomenon can be expected when responsiveness of some cells to field stimulation is reduced and the rate of current flow from adjacent cells is limited. Actually, similar desynchronized and delayed onsets could be mimicked by the gap junction inhibitor heptanol. Moreover, our previous data indicated that, in muscles showing desynchronized Ca^2+^ transients, non-phosphorylated Cx43 was significantly elevated in peripheral and intracellular regions, suggesting suppression of gap junction channel activity [6]. These results indicate that suppressed cell-to-cell coupling would cause a considerable delay in the onset of action potentials.

The decreased responsiveness is also reasonable because, in muscles showing delayed initiation of action potentials, those signals were, short-lasting, noisy and the rate of rise was slower than in the control. Those cells seem to be functionally compromised with inactivated Na^+^ channels, although blocking Na^+^ channels alone could not reproduce desynchronized action potentials within a field of view of microscope. It is reasonable to assume that both inhibition of gap channel activity and inactivation of Na^+^ channels occur in these muscles.

### Conclusions

In normal heart muscle, cell-to-cell propagation of action potentials in two-dimensional images is too fast to capture with a confocal microscope. However, under some pathophysiological conditions, the propagation is slowed sufficiently to be detected by optical imaging. The region of reduced conductivity can induce conduction block, action potential dispersion and/or T-wave alternans which lead to malignant arrhythmias. Further development of optical technologies for determination of Ca^2+^ responses and action potentials at the cellular level in tissue would provide valuable information for investigation of arrhythmias.

## Supporting Information

Figure S1 Ca^2+^ signals before (A) and after (B) pacing protocol in non-flowing Krebs solution. Left panel: Surface images of a cardiac muscle. Right panel: Time-based scan images. The muscle was stimulated at 2 Hz (arrows). Inset is a expanded time-based scan image showing delayed onset of Ca^2+^ transient.
(0.80 MB TIF)

Figure S2 Examples of alternating Ca^2+^ transients and membrane potential signals. A. Response to 1.43 Hz stimulation after Fig. 2A-c. B. Alternating amplitudes of Ca^2+^ and action potentials obtained from a different muscle. In this case, alternating large and small Ca^2+^ transients are attributed to immature action potentials with every second stimulus.
(2.31 MB TIF)

Movie S1 Ca^2+^ response at 1.67 Hz stimulation after the pacing protocol in non-flowing Krebs solution. This movie corresponds to the upper panel in Figure 2A-c.
(9.08 MB MOV)

Movie S2 Ratiometric action potential signals at 1.67 Hz stimulation after the pacing protocol in non-flowing Krebs solution. This movie corresponds to the lower panel in Figure 2A-c.
(8.82 MB MOV)
