# Supplementary figures and images for: Causes of Abnormal Ca2+ Transients in Guinea Pig Pathophysiological Ventricular Muscle Revealed by Ca2+ and Action Potential Imaging at Cellular Level
Source: PLoS One. 2009 Sep 21;4(9):e7069. doi: 10.1371/journal.pone.0007069 (PMC2740872; doi:10.1371/journal.pone.0007069)

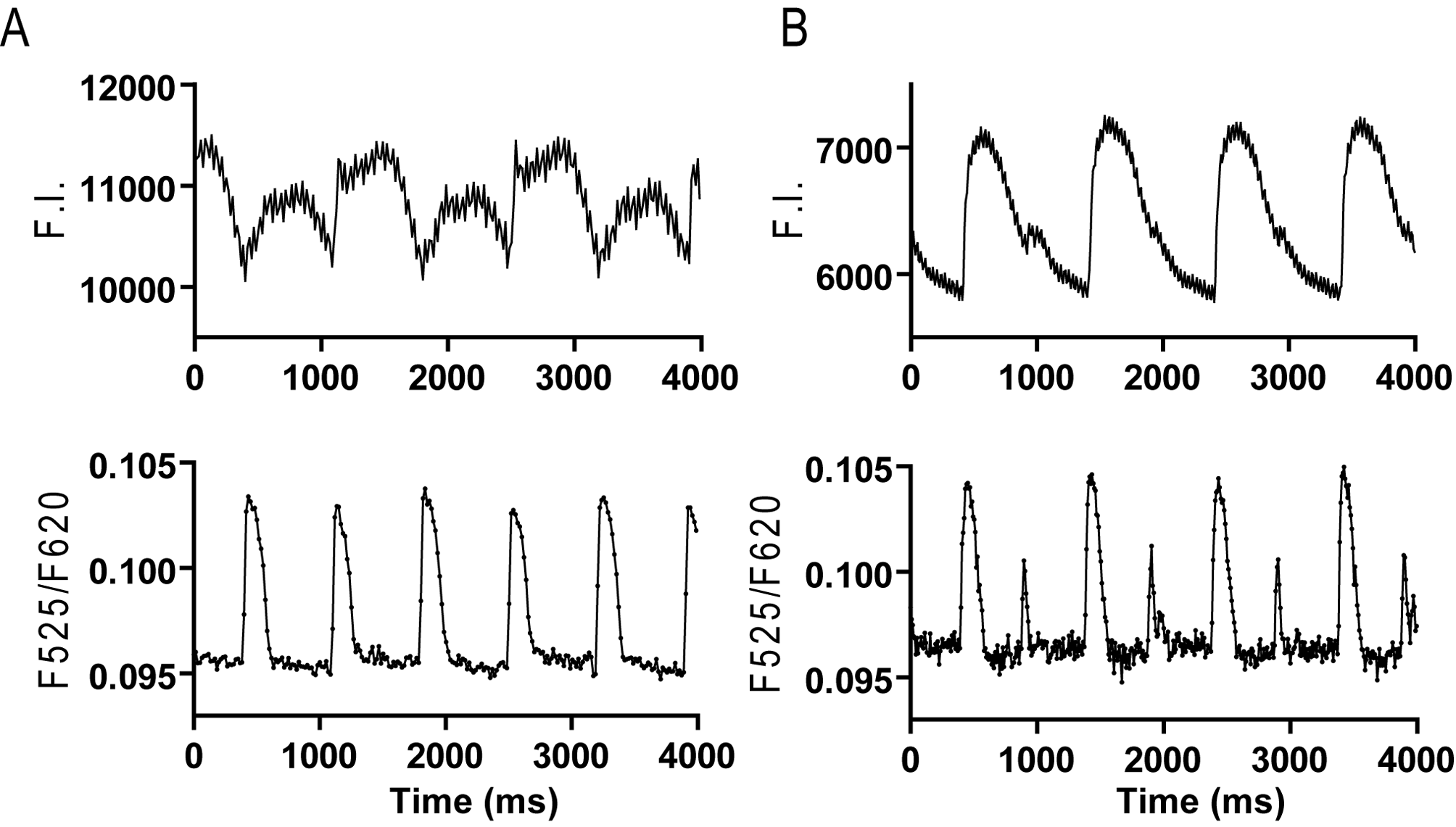

Supplement: Figure S2 — Examples of alternating Ca2+ transients and membrane potential signals. A. Response to 1.43 Hz stimulation after Fig. 2A-c. B. Alternating amplitudes of Ca2+ and action potentials obtained from a different muscle. In this case, alternating large and small Ca2+ transients are attributed to immature action potentials with every second stimulus. (2.31 MB TIF) [file pone.0007069.s002.tif]
